# Supplementary material for: Intrinsic brain dynamics in the Default Mode Network predict involuntary fluctuations of visual awareness
Source: Nat Commun. 2022 Nov 14;13:6923. doi: 10.1038/s41467-022-34410-6 (PMC9663583; doi:10.1038/s41467-022-34410-6)
Supplement: Supplementary file 1 — Supplementary Information [file 41467_2022_34410_MOESM1_ESM.pdf]

Supplementary Information for

Intrinsic brain dynamics in the Default Mode Network predict involuntary fluctuations of visual awareness

Dian Lyu[1,2], Shruti Naik[3], David K. Menon[1,4], Emmanuel A. Stamatakis[1,2*]

[1] University Division of Anaesthesia, University of Cambridge, Addenbrooke's Hospital, Hills Rd, CB2 0SP Cambridge, UK

[2] Department of Clinical Neuroscience, University of Cambridge, Addenbrooke's Hospital, Hills Rd, CB2 0SP Cambridge, UK

[3] Cognitive Neuroimaging Unit, INSERM, CEA, Université Paris-Saclay, NeuroSpin centre, 91191 Gif/Yvette, France

[4] Wolfson Brain Imaging Centre, University of Cambridge, Cambridge Biomedical Campus (Box 65), CB2 0QQ, Cambridge, UK

∗To whom correspondence should be addressed; E-mail: eas46@cam.ac.uk

This PDF file includes:

Materials and Methods

Figs.: sFig.1 to sFig.15

Tables: sTable 1 to 2

References *(1-23)*

Supplementary Figures

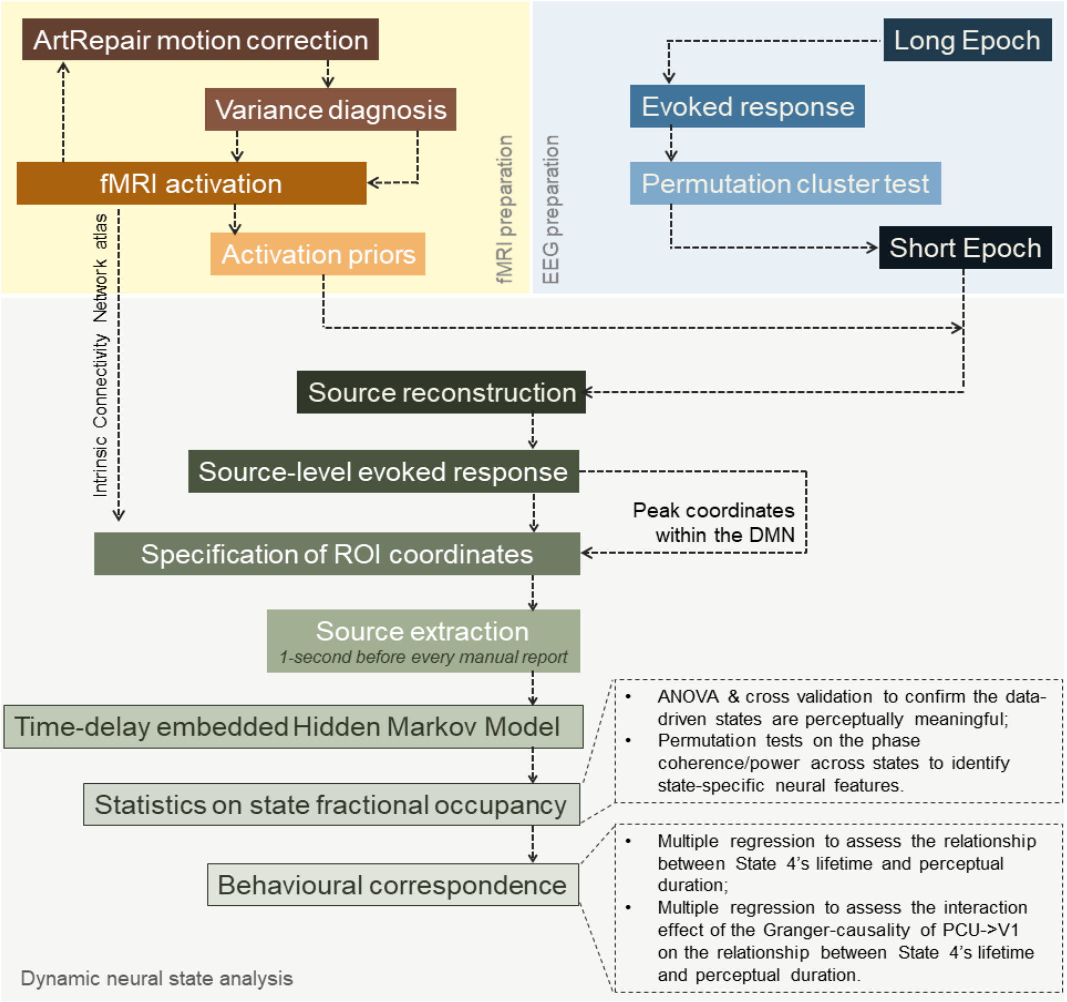

sFig. 1. Analysis pipeline for the simultaneous fMRI-EEG data. With preprocessed fMRI and EEG data, we conducted an initial data exploration (i.e., activation studies for fMRI and evoked-response analysis for EEG) by following standard neuroimaging analysis procedures. To ascertain the task-related regions at the network level, we adopted the Intrinsic-Connectivity-Network (ICN) atlas to evaluate fMRI activation patterns. Since this experiment adopted a continuous BR design where there is no explicit onset of a trial (while the offset is the manual indication of a perceptual transition), we constrained the trial to 1 second (s) by iteratively taking epochs from long to short durations (e.g., from 5 to 3 to 1 s) before a manual response, until we found significant evoked-potential differences between the BR and RPL conditions that were comparable to the previous literature. To initiate the dynamic neural state

analysis, we took the source signals (1 s) before every manual report from DMN peak coordinates, shown to be significantly involved in this task by source-level evoked response analysis. With the constrained temporal and spatial range, we set up the time-delay embedded Hidden Markov Model, which is a state-of-art analysis pipeline designed to discover the intrinsic dynamic patterns among a given set of brain regions. We searched for four common spatiotemporal patterns (i.e., states, with a micro-window of ~100 ms) that persist in all trials (of all conditions and participants). The states were estimated at each timepoint of a trial; hence, despite being transient, they were the most robust patterns recurring across time. To establish the states' perceptual relevance, we conducted ANOVA and cross validation analyses, using the states' presence to predict the perceptual type of the upcoming transition for each trial. Finally, to interpret the states, we extracted the spectral information from the states and conducted permutation tests across the states, which revealed the most distinctive neural features of each state. We further focused on certain neural features of the critical state to investigate how intrinsic DMN dynamics may have an influence on primary visual cortex.

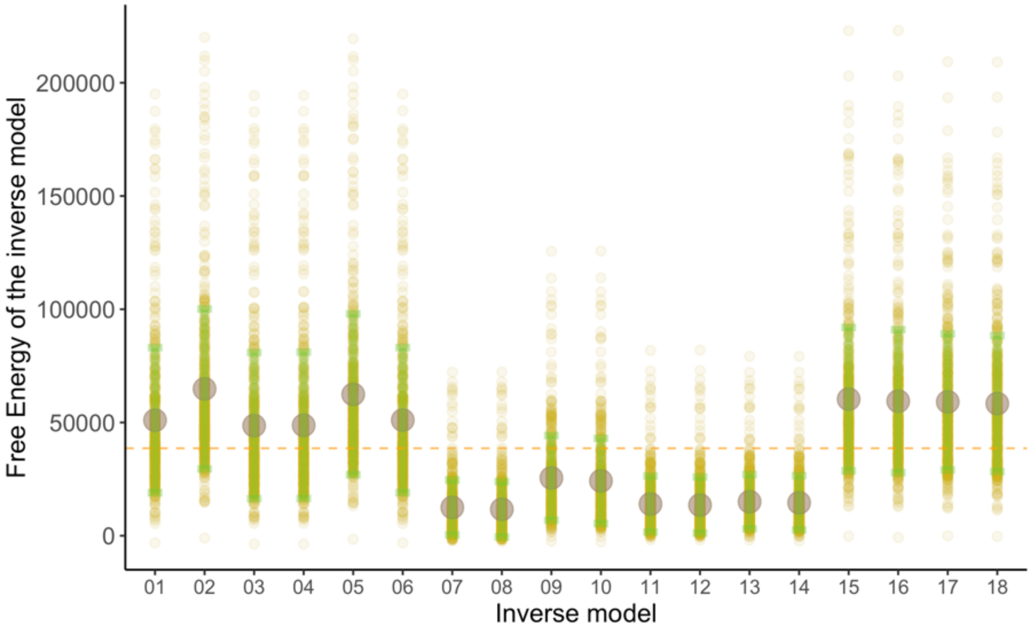

sFig. 2. Model comparison for different inverse modelling configurations. The free energy, which approximates the log model evidence, is used for model comparison. The inverse model (No. 2) with the highest free energy was chosen for further analyses[1,2]. The model specifications are described in sTable 1.

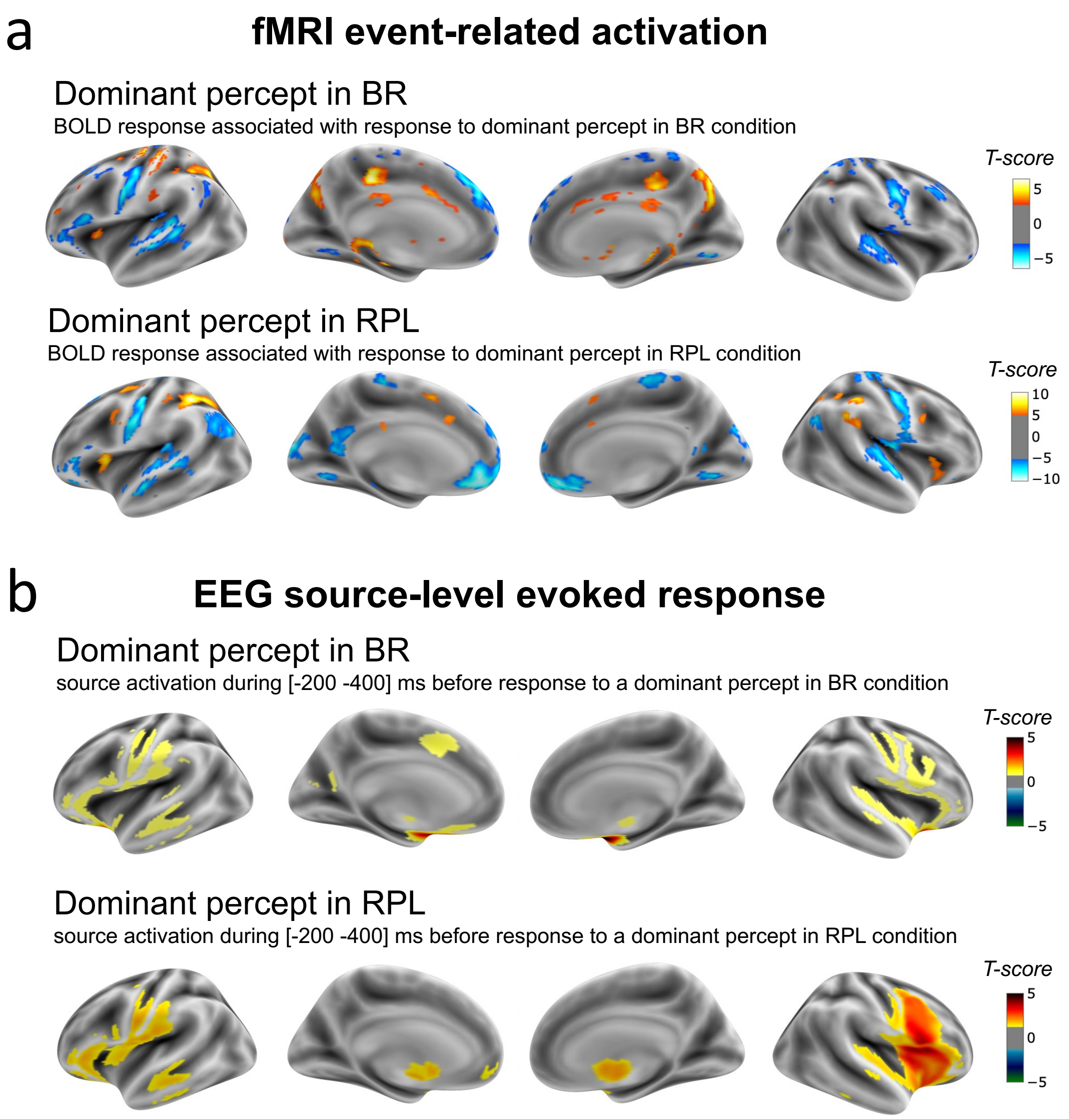

sFig. 3. (a) The fMRI event-related activation (without contrasts) for the dominant percept in both BR and RPL condition. (b) The EEG source-level evoked activation (without contrasts) during [-200 -400] ms before response to a dominant percept in both BR and RPL conditions. The brain map shows the brain regions with the top 10% of the T-scores.

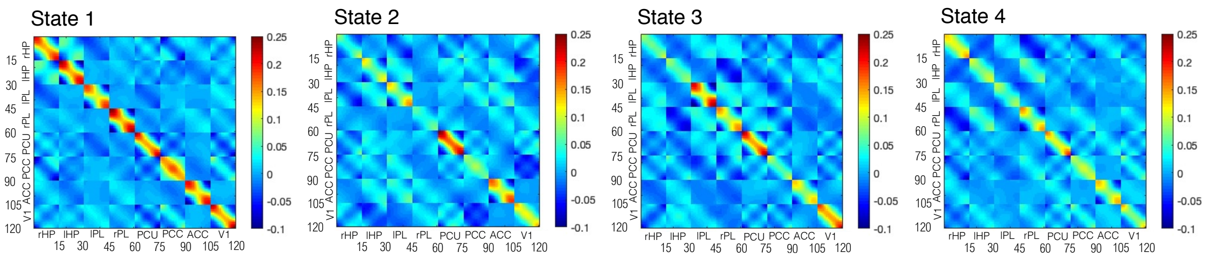

sFig. 4. The four states characterised by auto-covariance patterns (colour indicating correlation coefficient). Each value in the matrix indicates the lagged cross-covariance during a sampling window of 15 time-points for a region or between regions. The self-consistent auto-covariance pattern within each ROI emerges as a mosaic tile in the form of matrix visualisation. These "tiles" correspond to the regions: rHP, lHP, lPL, rPL, PCU, PCC, ACC and V1, in order.

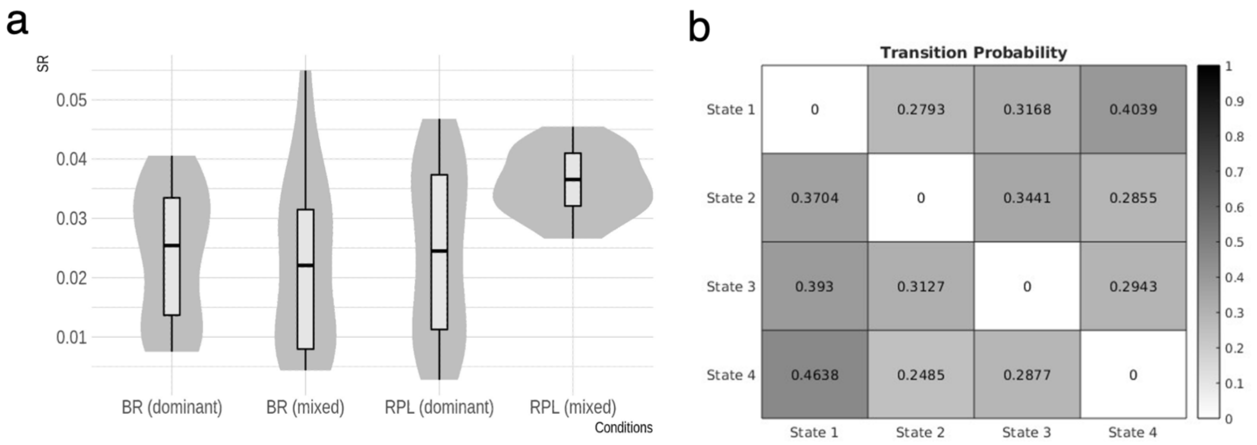

sFig. 5. (a) Switching rate (SR) of the states in the four experimental conditions (N = 20 per condition). There is no significant difference in the SR between conditions, except for the RPL (mixed) being faster than the rest. Boxplots and all other cases in this paper present the median, lower quartile and upper quartile of the data respectively at the middle, the lower and upper bound of the boxes. The data range is indicated by the whisker vertically centred at the box. (b) State transition probability across all conditions. This result showed that states were mixed well, as no states stagnated or dominated.

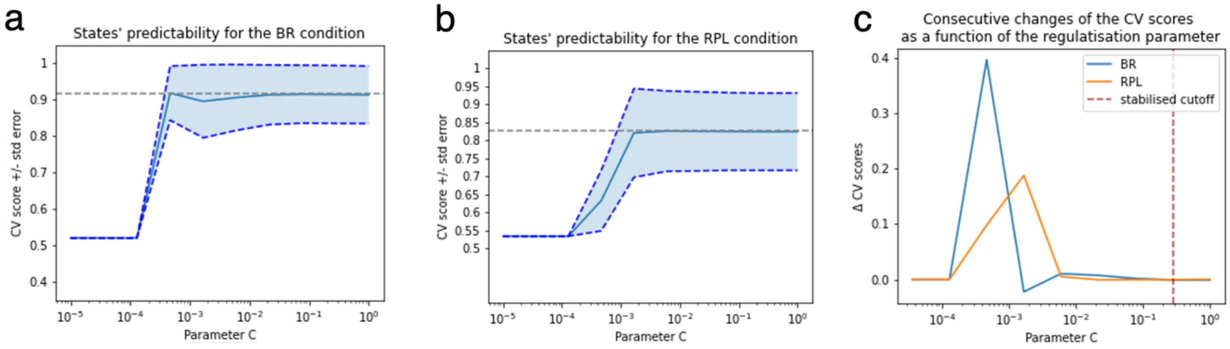

sFig. 6. Cross validation (CV) score improving and converging across a range of regularisation parameters, respectively for the data in the BR condition (a) and RPL condition (b). The CV score is the accuracy of using hidden states' FO to predict the type (mixed or dominant) of the upcoming transition in each trial. The middle line indicates the mean of accuracy across all shuffled runs. The two dashed lines surrounding the mean mark the range of one standard deviation. The grey dashed line parallel to the x-axis indicates the maximum accuracy the model has reached. (c) Consecutive changes of the CV scores as a function of regularisation parameters. This was used to identify the optimal range of the regularisation parameters, which was marked with the red dashed line. A two-sample *t*-test was conducted using the CV scores within the optimal range of the parameter C, for comparing the states' predictability between the BR and RPL conditions.

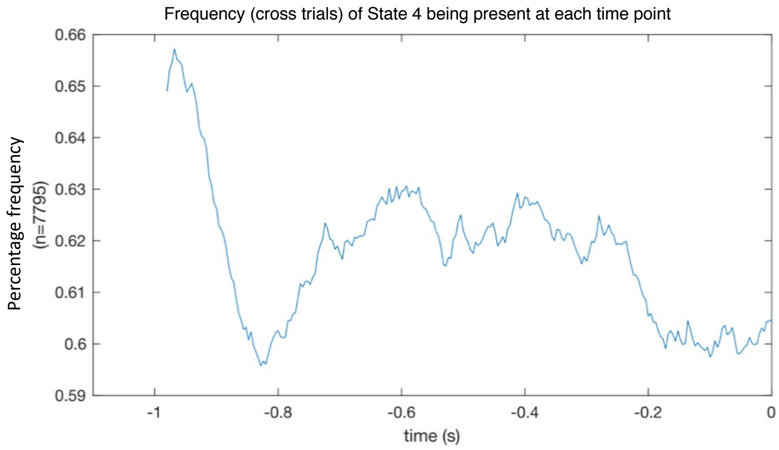

sFig. 7. The frequency (or proportion) cross trials of the State 4 being present at each time point in the BR (dominant) condition.

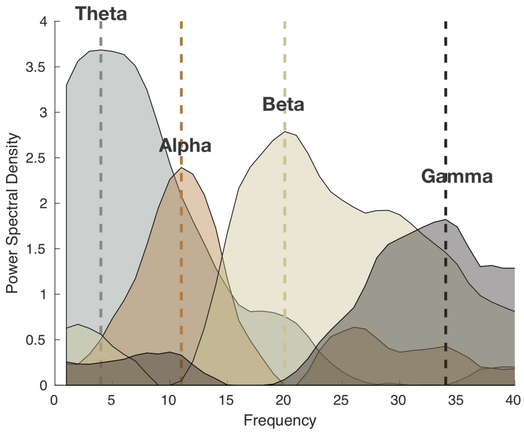

sFig. 8. Four components of the frequency spectrum for the spectral coherence of all the states. The four components were found with a non-negative matrix factorisation on the coherence matrix across all states and pairs of regions, which turned out to roughly correspond to classical frequency bands.

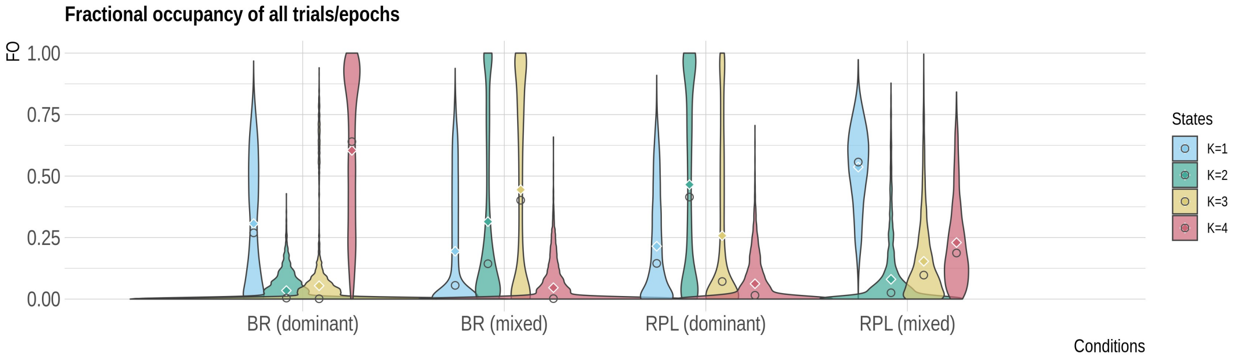

sFig. 9. State FO of all trials/epochs of all participants in the 4 conditions. The white rhombus mark indicates the mean and the grey circle mark indicates the median.

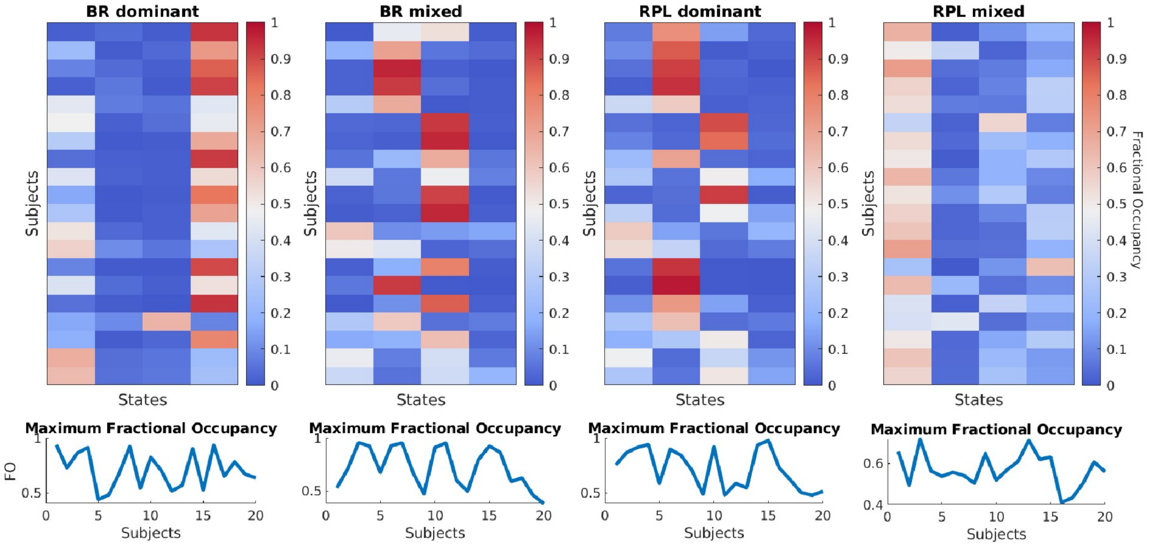

sFig. 10. State FO distribution over participants and across the 4 experimental conditions. The state FO distribution has noticeable individual difference, with some participants having higher representation of some states than others despite the variance caused by the condition difference. However, the condition-specific effect on the state FO surpasses the individual difference.

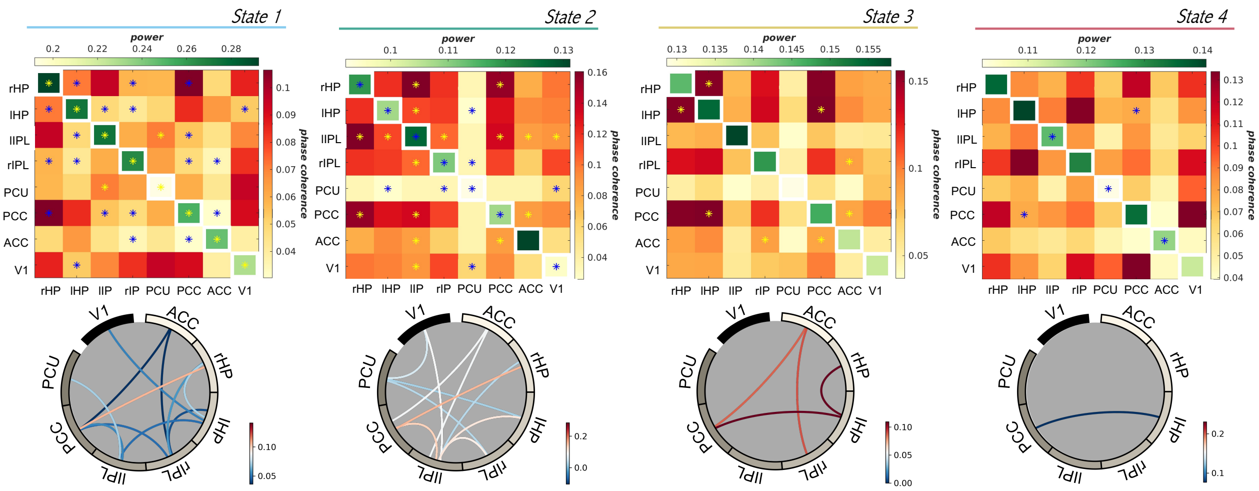

sFig. 11. Spectral information of the states in the theta band. Diagonal and off-diagonal values of the upper-triangle heatmaps respectively indicate the power and phase coherence of the ROIs. The asterisk on the heatmap indicates that the phase coherence (or power) in this state is significantly higher (yellow) or lower (blue) compared with the other states (one-tailed test with the 95% confidence interval by permutation). The circular bundle plots below highlight the significant connectivities, with red and blue respectively signifying higher and lower significance for the connectivity (colour bar indicating the phase coherence value).

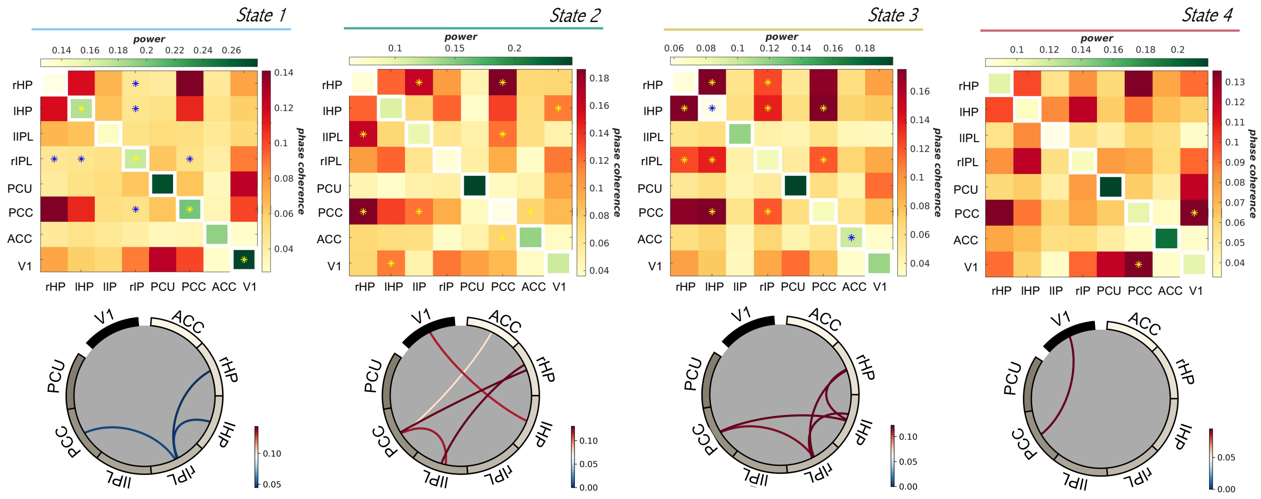

sFig. 12. Spectral information of the states in the beta band. Diagonal and off-diagonal values of the upper-triangle heatmaps respectively indicate the power and phase coherence of the ROIs. The asterisk on the heatmap indicates that the phase coherence (or power) in this state is significantly higher (yellow) or lower (blue) compared with the other states (one-tailed test with the 95% confidence interval by permutation). The circular bundle plots below highlight the significant connectivities, with red and blue respectively signifying higher and lower significance for the connectivity (colour bar indicating the phase coherence value).

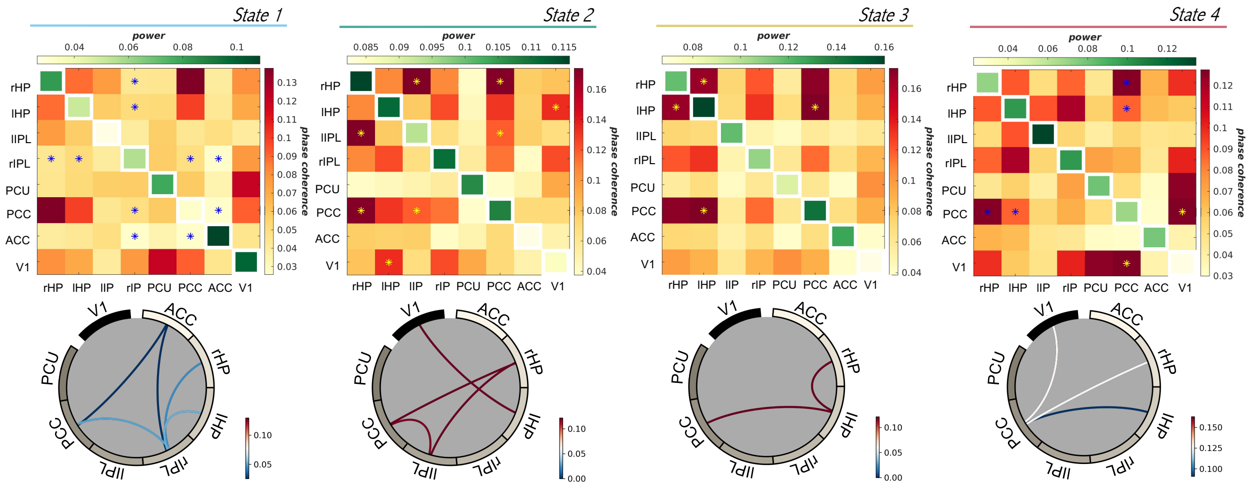

sFig. 13. Spectral information of the states in the gamma band. Diagonal and off-diagonal values of the upper-triangle heatmaps respectively indicate the power and phase coherence of the ROIs. The asterisk on the heatmap indicates that the phase coherence (or power) in this state is significantly higher (yellow) or lower (blue) compared with the other states (one-tailed test with the 95% confidence interval by permutation). The circular bundle plots below highlight the significant connectivities, with red and blue respectively signifying higher and lower significance for the connectivity (colour bar indicating the phase coherence value).

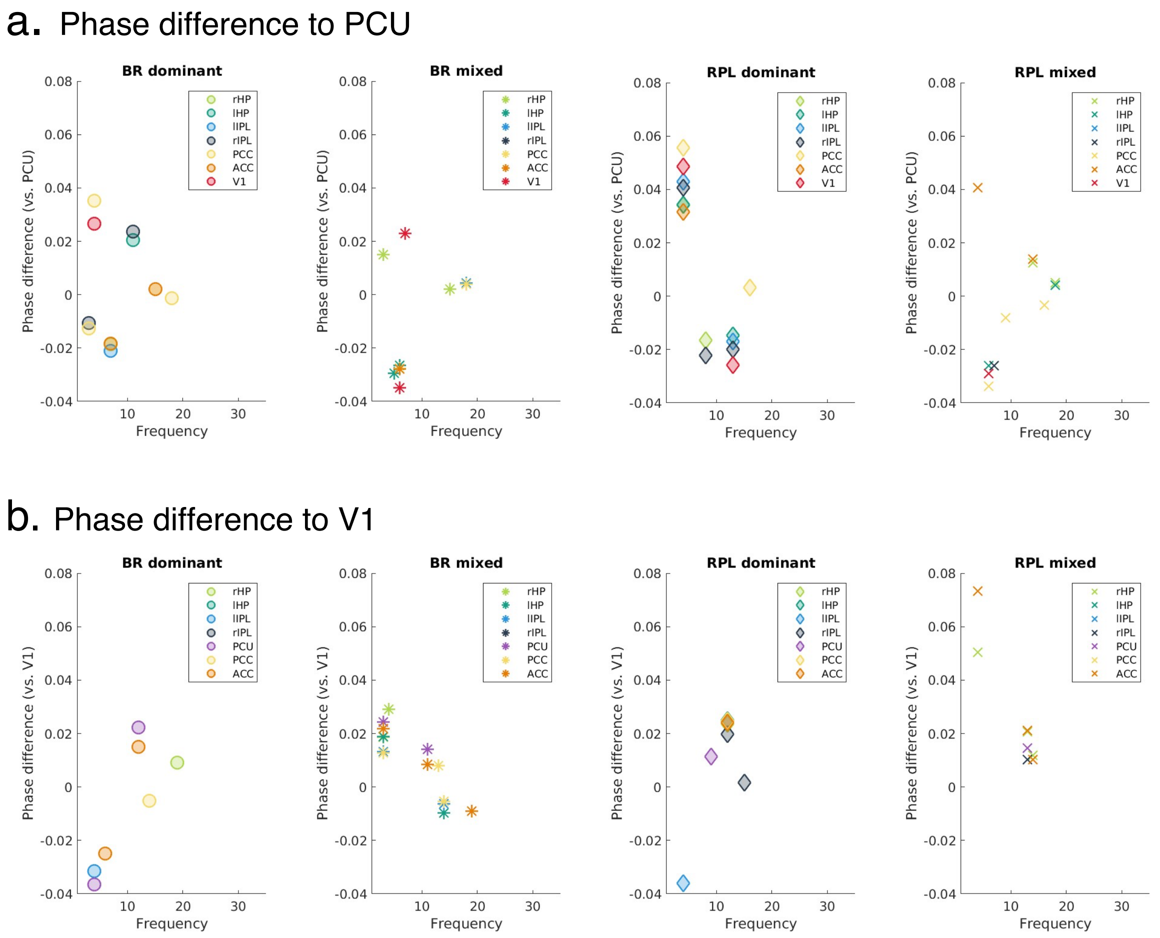

sFig. 14. Phase differences between the PCU (or V1) and the other regions of interest on original signals. The represented relationships have exceeded a 95% confidence interval of a null distribution (one-sample *t*-test, distribution estimated from trial variance for each condition).

(a) (b)

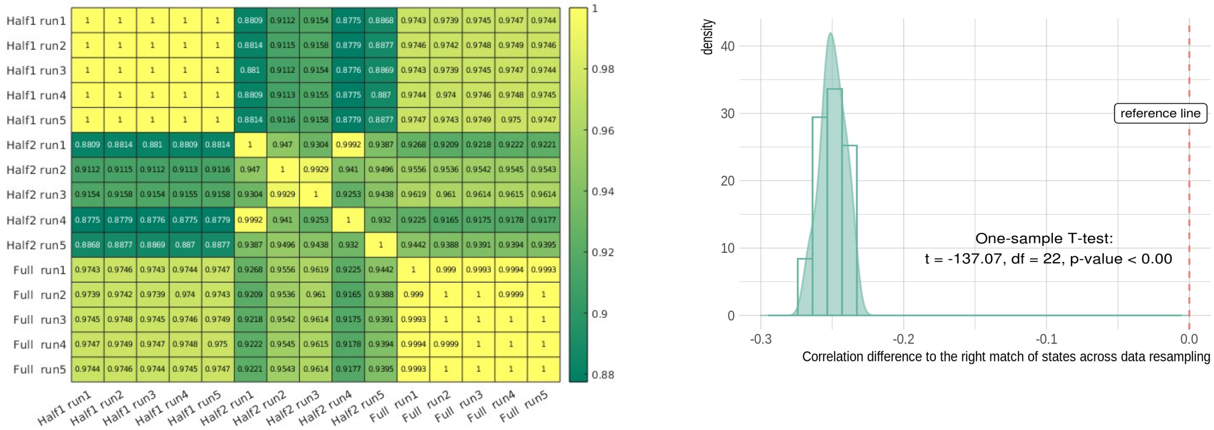

sFig. 15. (a) Reproducibility of the states, measured as Pearson correlation. Analyses were performed multiple times on half-split data and the full data. The random sampling for getting half of the subjects has been repeated 5 times. Besides, we also run the analyses on the full data for 5 times. The correlations between different runs are presented. For example, the number on row 1 and column 3 indicates the correlation between the states of the first-half data upon the first resampling and that of the first-half data of the first-half data upon the third resampling. (b) The consistency of the states between runs. Difference in correlation between matched state pairs and non-matched states pairs were calculated within each run, and the statistical testing was carried out on the group-level. The difference is significantly lower than zero ($p < 0.00$), suggesting that we have successfully identified the states in different runs.

Supplementary Tables

sTable 1: Inverse model specification for EEG source.

No.	Model	Use prior	Use hanning	Frequency (Hz)
1	IID	1	1	1-35
2	sLOR	1	0	1-35
3	IID	0	1	1-35
4	sLOR	0	1	1-35
5	sLOR	0	0	1-35
6	sLOR	1	1	1-35
7	sLOR	1	0	1-3.5
8	sLOR	0	0	1-3.5
9	sLOR	1	0	4-7.0
10	sLOR	0	0	4-7.0
11	sLOR	1	0	7.5-9.5
12	sLOR	0	0	7.5-9.5
13	sLOR	1	0	10-12.0
14	sLOR	0	0	10-12.0
15	sLOR	1	0	13-23
16	sLOR	0	0	13-23
17	sLOR	1	0	24-34
18	sLOR	0	0	24-34

sTable 2: Peak coordinates from which signals were extracted (and averaged within each ROIs) for constructing the HMM.

right para-hippocampal gyrus (rHP)	left para-hippocampal gyrus (lHP)	left inferior parietal lobule (lIPL)	right inferior parietal lobule (rIPL)	posterior cingulate cortex (PCC)	precuneus (PCU)	anterior cingulate cortex (ACC)	primary visual cortex (V1)
12 -36 -2	-22 0 -18	-58 -28 30	56 -38 38	8 -32 26	16 -72 30	4 50 -16	26 -88 26
22 -14 -28	-30 -2 -26	-44 -30 40	50 -42 48	-6 -24 30	12 -58 66	-8 32 -4	20 -82 30
16 -36 -4	-30 -4 -18	-58 -28 30	50 -34 30	-8 -56 14	4 -62 50	-6 40 -6	26 -90 26
26 -6 -20	-24 6 -22	-50 -30 52	56 -36 38	0 -24 24	10 -30 28	2 52 -14	24 -88 14
34 2 -20	-34 -2 -18	-54 -30 30	50 -32 30	-8 -48 14	-8 -24 28	-6 32 -12	26 -88 26
16 -38 -4	-24 6 -22	-58 -26 28	44 -32 60	10 -30 28	8 -58 42	-10 32 -4	
	-28 0 -16	-30 -4 -18	58 -36 40	-8 -24 28	12 -78 46	2 48 -16	
	-26 0 -20	-52 -42 48	58 -34 40	-8 -58 14	18 -70 32	-12 48 -2	
	-30 -2 -26	-42 -38 58	38 -46 38	0 -34 22	12 -52 48	2 50 -12	
	-26 6 -22	-58 -28 28	38 -44 38	6 -32 26	6 -58 50	-10 40 -2	
	-34 -12 -22	-52 -26 30	58 -32 40	-6 -24 30	4 -60 50	-6 40 -2	
	-22 -8 -16	-50 -28 30	40 -36 60	-10 -56 14		6 24 22	
	-24 -4 -22	-54 -26 30		-8 -26 30		8 38 -14	
	-24 -2 -16	-60 -26 28		-4 -56 12		-6 30 -6	
	-32 0 -22			-8 -48 14			
	-14 -36 -2			8 -32 26			

Supplementary References

1. López, J. D., Litvak, V., Espinosa, J. J., Friston, K. & Barnes, G. R. Algorithmic procedures for Bayesian MEG/EEG source reconstruction in SPM. *NeuroImage* **84**, 476–487 (2014).
2. Henson, R. N., Flandin, G., Friston, K. J. & Mattout, J. A Parametric Empirical Bayesian framework for fMRI-constrained MEG/EEG source reconstruction. *Hum. Brain Mapp.* **31**, 1512–1531 (2010).
