# Supplementary material for: Intrinsic brain dynamics in the Default Mode Network predict involuntary fluctuations of visual awareness
Source: Nat Commun. 2022 Nov 14;13:6923. doi: 10.1038/s41467-022-34410-6 (PMC9663583; doi:10.1038/s41467-022-34410-6)
Supplement: Supplementary file 3 — Reporting Summary [file 41467_2022_34410_MOESM3_ESM.pdf]

nature portfolio

Corresponding author(s): Dr Emmanuel A Stamatakis

Last updated by author(s): Sep 25, 2022

Reporting Summary

Nature Portfolio wishes to improve the reproducibility of the work that we publish. This form provides structure for consistency and transparency in reporting. For further information on Nature Portfolio policies, see our Editorial Policies and the Editorial Policy Checklist.

Statistics

For all statistical analyses, confirm that the following items are present in the figure legend, table legend, main text, or Methods section.

n/a	Confirmed	
☐	☒	The exact sample size (n) for each experimental group/condition, given as a discrete number and unit of measurement
☐	☒	A statement on whether measurements were taken from distinct samples or whether the same sample was measured repeatedly
☐	☒	The statistical test(s) used AND whether they are one- or two-sided<br>*Only common tests should be described solely by name; describe more complex techniques in the Methods section.*
☐	☒	A description of all covariates tested
☐	☒	A description of any assumptions or corrections, such as tests of normality and adjustment for multiple comparisons
☐	☒	A full description of the statistical parameters including central tendency (e.g. means) or other basic estimates (e.g. regression coefficient) AND variation (e.g. standard deviation) or associated estimates of uncertainty (e.g. confidence intervals)
☐	☒	For null hypothesis testing, the test statistic (e.g. F, t, r) with confidence intervals, effect sizes, degrees of freedom and P value noted<br>*Give P values as exact values whenever suitable.*
☐	☒	For Bayesian analysis, information on the choice of priors and Markov chain Monte Carlo settings
☐	☒	For hierarchical and complex designs, identification of the appropriate level for tests and full reporting of outcomes
☐	☒	Estimates of effect sizes (e.g. Cohen's d, Pearson's r), indicating how they were calculated

Our web collection on statistics for biologists contains articles on many of the points above.

Software and code

Policy information about availability of computer code

Data collection	Not relevant. The data is open source and can be obtained from the link: https://datadryad.org/stash/dataset/doi:10.5061/dryad.bf1b1. We have provided the link in the Data Availability section of the manuscript.
Data analysis	The code for analysis is made available from the link: https://github.com/Aubrey-Lyu/BR-project/. Dependent open-source toolboxes are (1) SPM12 (https://www.fil.ion.ucl.ac.uk/spm/software/spm12/); (2) SNPM (https://warwick.ac.uk/fac/sci/statistics/staff/academic-research/nichols/software/snpm/); (3) HMM-MAR (https://github.com/OHBA-analysis/HMM-MAR) and (4) Granger_cause_1 (https://www.mathworks.com/matlabcentral/fileexchange/59390-granger_cause_1)

For manuscripts utilizing custom algorithms or software that are central to the research but not yet described in published literature, software must be made available to editors and reviewers. We strongly encourage code deposition in a community repository (e.g. GitHub). See the Nature Portfolio guidelines for submitting code & software for further information.

Data

Policy information about availability of data

All manuscripts must include a data availability statement. This statement should provide the following information, where applicable:

- Accession codes, unique identifiers, or web links for publicly available datasets
- A description of any restrictions on data availability
- For clinical datasets or third party data, please ensure that the statement adheres to our policy

The preprocessed fMRI and EEG data that support the findings of this study are available in Dryad with the identifier doi:10.5061/dryad.bf1b1. The link has been provided in the Data Availability section in the manuscript.

Field-specific reporting

Please select the one below that is the best fit for your research. If you are not sure, read the appropriate sections before making your selection.

☒ Life sciences ☐ Behavioural & social sciences ☐ Ecological, evolutionary & environmental sciences

For a reference copy of the document with all sections, see nature.com/documents/nr-reporting-summary-flat.pdf

Life sciences study design

All studies must disclose on these points even when the disclosure is negative.

Sample size	The experiment involved 20 participants with two neuroimaging modalities (fMRI & EEG) recorded. Each participant underwent 10~14 scanning sessions, each containing five 42 second task blocks. This experimental design altogether generated 29785 epochs/trials for our analyses. We considered the study to be adequately powered before we decided to use this dataset. This study adopted a within-subject experimental design. For each participant, each experimental session/condition of rivalry or replay consisted of 5 consecutive 42-second blocks of continuous stimulus presentation followed by 12 seconds of rest, and 5 total blocks for each stimulus type. Each participant repeated the binocular rivalry and replay sessions 5-7 times to increase replicability, which generated about 240 scanning sessions and 29785 trials in total for analysis (about 6 scanning sessions and 327 trials on average for each participant in each experimental condition). We consider this study is adequately powered for a within-subject analysis of the standard neuroimaging processing (i.e., fMRI activation and EEG evoked-response analyses). We also conducted a post-hoc power calculation with the software G*power (https://stats.idre.ucla.edu/other/gpower/). The detailed results can be seen in our response to Reviewer 1.
Data exclusions	For the EEG data, bad channels and epochs were identified and excluded from further analysis. Noise contaminated electrodes were rejected following visual inspection of a histogram displaying average power for each electrode. Bad epochs were defined as periods when the mean global field power exceeded five standard deviations above the mean (Jamison et al., 2015, J. Neurosci; Roy et al., 2017, NeuroImage). For the fMRI data we applied additional checks as follows: volumes with > 3mm displacement compared to the previous volume were considered to be contaminated by fast motion. Overall, bad volumes comprised on average 5.12% (STD=7.05%), and in the worst case 34% (44 volumes out of 129) of a person's data in one scanning session. As a result, no participant's data were excluded from our subsequent analyses.
Replication	The reproducibility was not formally tested for fMRI activation and EEG evoked response analyses. The sensor-level EEG evoked response analyses were independently carried out twice by Dian Lyu and Shruti Naik and generated the same result. The source-level EEG evoked activation analyses were estimated twice, first with the statistical parametric mapping (SPM) and then with statistical non-parametric mapping (SnPM), i.e. permutation approach. Both analyses generated similar spatial localisations. Only SnPM results were reported because we did not confirm the normality of the source signals.<br>The reproducibility of our main findings were formally tested and reported. The data-driven neural states were reproduced by running the same algorithms 5 times on the same full datasets and 10 more times on different halves of the datasets. The halving of the data was produced by randomly selecting half of the data so that data combination was different for each resampling.
Randomization	The experiment has a within-subject design, by which the participant experienced the scanning sessions of all conditions. The stimulus for the control condition ("replay") was designed to approximate the experience of binocular rivalry where the perceptual transition happens randomly; therefore, the duration of each red or green epoch was randomly drawn from durations reported by that subject during the rivalry condition. In addition, for each participant, percept durations for the replay condition were "played back" based on a previous session of rivalry for the same participant. In some cases, multiple sessions of rivalry were recorded prior to a replay session; the rivalry session chosen for playback was then randomly selected from them.
Blinding	Blinding is not applicable to this study because there is no group allocation.

Reporting for specific materials, systems and methods

We require information from authors about some types of materials, experimental systems and methods used in many studies. Here, indicate whether each material, system or method listed is relevant to your study. If you are not sure if a list item applies to your research, read the appropriate section before selecting a response.

Materials & experimental systems

n/a	Involved in the study
☒	☐ Antibodies
☒	☐ Eukaryotic cell lines
☒	☐ Palaeontology and archaeology
☒	☐ Animals and other organisms
☐	☒ Human research participants
☒	☐ Clinical data
☒	☐ Dual use research of concern

Methods

n/a	Involved in the study
☒	☐ ChIP-seq
☒	☐ Flow cytometry
☐	☒ MRI-based neuroimaging

Human research participants

Policy information about studies involving human research participants

Population characteristics	Twenty-three healthy human subjects (17 male, 6 female, median age 23 years) participated in the study. 20 of them were reported in the previous publications (Jamison et al., 2015, J. Neurosci; Roy et al., 2017, NeuroImage) and are available from the Drayed data repository.
Recruitment	We have no access to this information.
Ethics oversight	The Institutional Review Board at the University of Minnesota.

Note that full information on the approval of the study protocol must also be provided in the manuscript.

Magnetic resonance imaging

Experimental design

Design type	Even-related design
Design specifications	For each participant, each experimental session/condition of rivalry or replay consisted of 5 consecutive 42-second blocks of continuous stimulus presentation followed by 12 seconds of rest, and 5 total blocks for each stimulus type. Each participant repeated the binocular rivalry and replay sessions 5-7 times to increase replicability.
Behavioral performance measures	For behavioural response, the participant pressed one of the three buttons, each indicating a type of percept (green, red or mixed). The interval between every two percepts indicated the length of the previous percept. The mean duration of a percept across task blocks was used as a statistic of perceptual duration for that subject.

Acquisition

Imaging type(s)	Functional, structural MRI
Field strength	3 Tesla
Sequence & imaging parameters	The whole-brain BOLD functional data were acquired using a typical GE-EPI pulse sequence (FA = 90◦, TR = 2200 ms, TE = 30 ms, 3 mm isotropic voxels, 36 axial slices, with fat saturation pulse).
Area of acquisition	Whole brain.

Diffusion MRI ☐ Used ☒ Not used

Preprocessing

Preprocessing software	The data were preprocessed by the data distributor using the default preprocessing pipeline with the SPM12 software.
Normalization	Non-linear SPM 12 spatial normalisation algorithm
Normalization template	MNI
Noise and artifact removal	Six head-movement (translation and rotation) parameters and their first eigenvectors extracted from white matter and CSF signals were treated as non-neuronal noise and were regressed out for the subsequent GLM statistical testing. In addition, motion-corrupted volumes identified by ArtRepair were de-weighted during the GLM modeling. Slow drifts in the fMRI signals were filtered out (cutoff = 128 seconds), after which, the temporal autocorrelation was estimated from the residuals and was removed. Also see volume censoring.
Volume censoring	We conducted the volume censoring with the default setting of the ArtRepair toolbox. The criterion is described as follows: the main GLM for statistical inferences was estimated twice, once with the ArtRepair and once without. The ArtRepair treatment to motion-corrupted volumes is to de-weight the contribution of them in a GLM estimation. To determine if the treatment was necessary, a global quality metric of the range of contrast estimates over every voxel within the standard brain mask was provided. For good contrast estimates, the mean of the contrasts should be near zero and the mean of the ResMS should be small (https://cibsr.stanford.edu/tools/human-brain-project/artrepair-software.html). If the post-hoc global quality metrics suggested that the original contrast estimate was actually better, no ArtRepair treatment would be applied. Overall, repaired volumes comprised on average 5.12% (STD=7.05%), and in the worst case 34% (44 volumes out of 129) of a person’s data in one scanning session.

Statistical modeling & inference

Model type and settings	For fMRI and EEG evoked-response activation analyses, the standard SPM12 approach was used, which involves a univariate and mixed model design, estimated for the whole-brain. As a mixed model design, the task-related activation was estimated independently for each subject in the first-level analysis. Then the contrasted beta (coefficient) images were used as dependent variables for the group-level analyses.

Effect(s) tested	The experiment has a 2-by-2 (2 types of percepts * 2 ways of generating the percepts) design. The contrasts between the 2 types of percepts and between the 2 ways of percept generation were conducted within individuals with a mixed factorial design, corrected for the session and block effects. In the first-level model, the 2 types of percepts were considered as repeated measures (or within-group factor) because they were measured within the same session; while the 2 ways of percept generation were considered as independent measures (or between-group factor) as they were measured in different scanning sessions. The main effects/contrasts of the two factors and the interaction effect between them were then examined with one-sample T tests on the group level.

Specify type of analysis: ☒ Whole brain ☐ ROI-based ☐ Both

Statistic type for inference (See Eklund et al. 2016)	The primary voxel-wise threshold was set at P = 0.001 (uncorrected), which was followed by a cluster-wise threshold of P = 0.05 (FWE-corrected).
Correction	P = 0.05 (FWE-corrected)

Models & analysis

n/a	Involved in the study
☐	☒ Functional and/or effective connectivity
☒	☐ Graph analysis
☐	☒ Multivariate modeling or predictive analysis

Functional and/or effective connectivity	No connectivity analysis was conducted for the fMRI data. But we used connectivity analyses for the EEG data and the common statistical principles apply. As an index of functional connectivity, we used phase coherence, which was decomposed from cross-covariance patterns (with a sliding window of 15 time-points) between ROIs during the epoch (with 250 time-points in total). As an index of effective connectivity, we used granger causality, performed on the temporal domain for the source signals (with 250 sample points).
Multivariate modeling and predictive analysis	No multivariate modelling or predictive analysis was performed on the fMRI data. But we have performed Hidden Markov model (multivariate autoregressive model) and cross validation for EEG data, and common statistical principles apply. For the Hidden Markov model (multivariate autoregressive model), the independent variable was the timeseries of the ROIs of all the epochs. The ROIs were identified from the significant regions in the activation analyses. The features extracted were four cross-covariance patterns (i.e., latent neural states) constructed within and between ROIs across time. For the cross validation, the predictors were the fractional occupancy of the four latent neural states and the dependent variable was the percept type for the upcoming perceptual transition.
